# Immunotherapy in Neuroendocrine Neoplasms: A Diamond to Cut

**DOI:** 10.3390/cancers16142530

**Published:** 2024-07-13

**Authors:** Esmeralda García-Torralba, Esther Garcia-Lorenzo, Bernard Doger, Francesca Spada, Angela Lamarca

**Affiliations:** 1 Department of Medical Oncology, Hospital Universitario Morales Meseguer, 30008 Murcia, Spain; esmeralda.garciat@um.es; 2 Department of Medicine, Medical School, University of Murcia, 30001 Murcia, Spain; 3 IMIB-Arrixaca, 30120 Murcia, Spain; 4 START Madrid-FJD, Early Phase Clinical Trials Unit, Fundación Jiménez Díaz University Hospital, 28040 Madrid, Spain; 5 European Institute of Oncology, European Institute of Oncology (IEO) IRCCS, 20141 Milan, Italy; francesca.spada@ieo.it; 6 Department of Oncology, OncoHealth Institute, Fundación Jiménez Díaz University Hospital, 28040 Madrid, Spain; 7 Department of Medical Oncology, The Christie NHS Foundation, Manchester M20 4BX, UK; 8 Division of Cancer Sciences, University of Manchester, Manchester M13 9PL, UK

**Keywords:** neuroendocrine neoplasms, immunotherapy, immune biomarkers

## Abstract

**Simple Summary:**

The development of new treatments for patients with neuroendocrine neoplasms (NENs) is imperative. Immunotherapy has shown efficacy in various neoplasms, such as small cell lung cancer and Merkel cell carcinoma. Although immunotherapy’s effectiveness is more limited in NENs, combining immune checkpoint inhibitors with other therapeutic strategies like chemotherapy or targeted therapies could improve outcomes. Additionally, identifying predictive immune biomarkers could enhance patient selection. Our objective was to review the current evidence of immunotherapy in NENs, covering efficacy results and potential predictive biomarkers.

**Abstract:**

A raise in the incidence of NENs is expected. Therefore, the identification of new therapeutic strategies, such as immunotherapy, remains crucial. To date, immune checkpoint inhibitors as monotherapy have shown modest activity in unselected NENs. Although immunotherapy combos (plus another immune agents or chemotherapy, among others) are potentially more active than single agents, this has not been uniformly confirmed, even in high-grade NENs. Other immunotherapeutic strategies under development include bispecific antibodies, targeting specific tumor antigens like DLL3, and cell therapy. Currently, no predictive immune biomarkers are available to guide clinical decisions. A comprehensive tumor molecular profiling approach needs to be developed for the selection of patients with NEN who could potentially benefit from immunotherapy. Ideally, clinical trials should incorporate this tumor molecular profiling to identify predictive biomarkers and improve efficacy. Achieving this goal requires an international collaborative effort.

## 1. Introduction

Neuroendocrine neoplasms (NENs) are a group of rare epithelial tumors that originate from the neuroendocrine system, and they are widely distributed throughout the body [1]. The global occurrence of NENs is on the rise, affecting all anatomical sites, histological grades, and stages [2]. Although a biological and clinical heterogeneity is the rule for these malignancies, they usually debut as low-grade, non-functioning, advanced-stage neoplasms (with 40–50% of patients having distant metastases at diagnosis) [3].

Currently, the standard treatment for well-differentiated NENs includes surgery, local ablative treatments, agents targeting somatostatin receptors, peptide receptor radionuclide therapy (PRRT), and therapies directed at specific targets [4]. However, emerging treatment approaches, such as exploring immunotherapy, are under investigation for managing NENs.

Immune evasion by tumors plays a crucial role in tumorigenesis and progression. Tumor cells employ various mechanisms to suppress the immune response, with a primary focus on reversing the inhibition of adaptive immunity through a blockade of T cell checkpoint pathways [1]. In this context, immunotherapy has emerged as a way to enhance a patient’s own immune system in combating cancer.

The identification of immune regulatory molecules, such as the PD1/PD-L1 axis, has led to the revolution of immunotherapy in cancer. Several phase III trials using monoclonal antibodies targeting the interaction between the PD-1 checkpoint and its ligands (PD-L1 and PD-L2), or the CTLA-4 checkpoint molecule, have demonstrated durable responses across different histologies [5].

While immunotherapy has transformed the standard of care in many cancers, its efficacy varies widely among histologies and even among patients with the same type of tumor. Moreover, immunotherapy can induce autoimmune side effects, including potentially life-threatening toxicities such as pneumonitis and myocarditis. Some of these side effects can also become chronic (e.g., endocrine disorders), significantly impacting patients’ quality of life. This underscores the need for new predictive and prognostic factors to identify patients who are most likely to benefit from these new therapies [6]. 

Like many carcinomas of various origins, clinical trials investigating immunotherapy have been conducted in NENs. This review focuses on the clinical efficacy of immunotherapies in NENs and discusses the role of current biomarkers in predicting responses to immune checkpoint inhibitors.

## 2. Immunotherapy in Neuroendocrine Neoplasms: Current Evidence of Efficacy

Several monotherapies, such as pembrolizumab, avelumab, spartalizumab, and toripalimab, have been evaluated in patients with gastroenteropancreatic neuroendocrine neoplasms (GEP-NENs), yielding inconsistent results (Table 1). 

### 2.1. Pembrolizumab

Pembrolizumab monotherapy was evaluated in two different KEYNOTE trials. The non-randomized phase 1b KEYNOTE-028 “basket” trial assessed pembrolizumab in a large cohort of patients with advanced solid tumors and positive PD-L1 (≥1% membranous expression in tumor or stromal cells). Among them, 25 patients with locally advanced or metastatic well/moderately differentiated gastrointestinal NENs (GI-NENs) and 16 pancreatic NETs (PanNETs) were enrolled. The objective response rate (ORR) was 12.0% and 6.3%, respectively. Interestingly, the median duration of response in the carcinoid cohort was 9.2 months, while it was not reached in the PanNET cohort. Overall survival at the first-year evaluation mark was 87% [7].

The phase 2 KEYNOTE-158 trial enrolled 107 previously treated patients with advanced, unresectable, or metastatic well-differentiated NENs (*n* = 40 PanNETs, *n* = 43 GI-NETs, and *n* = 14 lung NETs). Among them, 15.9% had PD-L1-positive tumors (combined positive score ≥ 1). The efficacy was limited, with an ORR of 3.7%. However, partial response (PR) was observed in 3 patients with PanNETs and in 1 patient with rectal NET [8].

### 2.2. Avelumab

Although evidence on the efficacy of avelumab in NENs is limited, the phase II AVENEC trial (ClinicalTrials.gov (accessed on 4 May 2024) NCT 03352934) reported an ORR of 6.9% in high-grade NENs from different origins (72.4% GEP). Promising results have been observed in patients with Merkel carcinoma (MCC) [9,19,20]. Avelumab’s activity and efficacy have been extensively studied in the large JAVELIN program, which included both pretreated metastatic MCC patients and those naïve to systemic therapy.

For the cohort of patients treated with avelumab as first-line therapy (*n* = 116), the ORR was 39.7% and the median OS was 20.3 months [21]. Among patients whose disease had progressed after more than one prior line of therapy (*n* = 88), the 60-month OS rate was 26%, with a median OS of 12.6 months [22]. The treatment was generally well tolerated. Based on the data from the JAVELIN Merkel 200 trial, avelumab is currently considered the standard of care for metastatic MCC and the only immunotherapeutic agent universally approved for systemic treatment across all NENs. 

### 2.3. Spartalizumab

Spartalizumab was evaluated in a phase II trial that enrolled patients with well-/poorly differentiated NENs, including GI-NETs (*n* = 32), PanNETs (*n* = 33), thoracic NETs (*n* = 30), and gastroenteropancreatic neuroendocrine carcinomas (GEP-NECs, *n* = 21). The primary endpoint of the study (prespecified activity threshold of ≥10% in the NEN group) was not reached, with a global ORR of 4.8% in the GEP-NEC cohort and 7.4% in the NET group, resulting in the study being considered negative. Notably, a higher ORR was observed in the thoracic NET group (16.7%) compared to the GI (3.1%) and pancreatic (3.0%) cohorts, suggesting a potentially more active role of spartalizumb in thoracic NETs [10]. 

Patients with ≥1% PD-L1 expression showed a higher ORR, and PD-L1 overexpression was more common in GEP-NECs than in GEP-NENs. Another identified predictive factor was the presence of ≥1% CD8+ cells at baseline [10]. 

### 2.4. Toripalimab

The efficacy of the humanized IgG4 anti-PD-1 toripalimab was evaluated in a phase 1b trial involving 40 patients with advanced NENs (Ki-67 ≥ 10%) who progressed after first-line treatment. The ORR was 20% (8 partial responses and 6 stable diseases) and the median duration of response was 15.2 months [11]. As predictors of greater benefit, the authors suggested PD-L1 ≥ 10% (ORR 50.0% vs. 10.7%), a tumor mutational burden (TMB) ≥ 9.9 mut/Mb (ORR 75.0% vs. 16.1%), and/or high microsatellite instability (MSI-H) [11].

## 3. Dual Therapy

Based on the limited activity of monotherapy strategies mentioned above, combination strategies (immunotherapy combos and combinations of immunotherapy with targeted therapies or chemotherapy) were explored (Table 1).

### 3.1. Nivolumab Plus Ipilimumab

The combination of ipilimumab and nivolumab has been extensively evaluated in NENs. The prospective, multicenter, open-label, phase II “basket” trial DART SWOG 1609 evaluated the activity and safety of ipilimumab plus nivolumab across multiple rare tumor cohorts. A total of 32 patients with any grade of non-pancreatic primary localization NENs (45% GI-NENs and 18% lung NENs) were included. A 44% ORR was observed in patients with NECs (*n* = 18) compared to 0% in patients with low- and intermediate-grade NENs (*n* = 14) [12].

Similarly, in the subgroup analysis of the CA209-538 clinical trial for rare cancers, 29 patients with advanced NENs (excluding patients with small cell lung cancers) were included. This multicenter, open-label, phase II study was conducted in five centers in Australia. Overall, 90% of patients had intermediate- (45%) or high-grade (45%) NENs: 39% lung NENs and 23% GEP-NENs. The ORR was 43% in patients with PanNENs and 33% in those with atypical lung carcinoids [13].

Preliminary results from non-comparative, phase II, NIPINEC trial (ClinicalTrials.gov (accessed on 4 May 2024) NCT 03591731) were recently reported (ESMO annual meeting, 2021). This study evaluated 85 patients with refractory (24% of whom had asymptomatic brain metastases) GEP-NECs (*n* = 42 were treated with nivolumab, and *n* = 43 with nivolumab plus ipilimumab). The ORR at 8 weeks was 7.2% with the monotherapy and 14.9% with the combination. Median PFS was 1.8 months and 1.9 months, respectively [23].

### 3.2. Durvalumab Plus Tremelimumab

The DUNE phase II trial has a design similar to the previously mentioned phase II trial with spartalizumab as a single agent and included 123 patients with NENs divided into four different cohorts: (1) lung carcinoids (*n* = 27), (2) grade 1-2 GI-NETs (*n* = 31), (3) grade 1-2 PanNETs (*n* = 32), and (4) grade 3 GEP-NENs (*n* = 33, 91% NECs). The 9-month clinical benefit rate (CBR: complete/partial response and stable disease) was the primary endpoint for the cohorts 1–3, and it was 25.9%, 35.5%, and 25%, respectively. A CBR of 36% was observed at a median follow-up of 10.8 months in the PanNEN cohort. The 9-month overall survival (OS) rate was the primary endpoint for the cohort 4 [14,24]. 

### 3.3. Nivolumab Plus Platinum-Doublet Chemotherapy

It has been proposed that chemotherapy could increase the tumor mutational burden and immune tumor infiltration, thereby making immunotherapy more effective. Final survival results from the NICE-NEC trial have been recently reported [15]. They enrolled 38 patients with GEP or unknown origin grade 3 NENs who had not been previously treated. Overall, 68.4% had NECs and 65.8% had high Ki-67 (>55%). After a median follow-up of 18.6 months, the ORR was 54.1% and the median PFS was 5.7 months. The 12-month OS rate was 46.7% for high Ki-67 and 59.3% for low Ki-67, respectively [15].

### 3.4. Nivolumab Plus Temozolamide

Nivolumab combined with temozolomide was explored in a phase II, multi-cohort, single-center, non-randomized trial [16]. This study included 28 patients with NENs (71% NET, 29 NEC) from different primary locations (*n* = 11 GI-NENs, *n* = 11 lung NENs, *n* = 3 PanNENs, *n* = 1 head and neck NEN and *n* = 2 unknown NENs). The ORR was 32.1% (*n* = 9), with a significant difference in responses by primary tumor location (lung vs. others, *p* = 0.020). The median PFS was 8.8 months. Interestingly, patients who achieved a partial response had lower levels of LAG-3-expressing T cells [16].

### 3.5. Atezolizumab Plus Bevacizumab

The combination of atezolizumab and bevacizumab was evaluated in an open-label, phase 2 basket trial with 8 parallel rare tumor cohorts [17]. A total of 40 patients with advanced grade 1 to 2 NENs (20 with PanNENs and 20 with extra-pancreatic NENs (ePanNENs)) were included. An ORR was observed in 4 (20%) patients with PanNENs and 3 (15%) patients with ePanNENs. With a safe profile, the median PFS was 14.9 months and 14.2 months, respectively [17].

### 3.6. Spartalizumab Plus LAG525

Spartalizumab and LAG525 are monoclonal antibodies targeting PD-1 and LAG-3, respectively, which have shown synergistic antitumor activity in preclinical models. In a phase II study of advanced solid tumors, the primary endpoint was reached in the GEP-NEN cohort, with an 86% CBR at 24 weeks [25].

## 4. Other Immunotherapy Approaches

Additional immunotherapy approaches under investigation include bispecific antibodies or bispecific T-cell engagers, which target both CD3 on lymphocytes and specific tumor antigens like DLL3 simultaneously [26]. Oncolytic viruses are utilized to enhance the responsiveness of immunologically ‘cold’ tumors to immune checkpoint blockades [26]. Further details are provided in Section 8.

## 5. Combination of Surgical Procedures and Immunotherapy in Neuroendocrine Neoplasms

The integration of surgical procedures and immunotherapy in the treatment of NENs is an evolving field, aimed at improving patient outcomes through a multimodal approach. Combining surgery with immunotherapy can potentially induce an upgraded immune response. Surgical debulking can reduce the immunosuppressive tumor microenvironment, making residual tumors more susceptible to immunotherapy. Additionally, surgery may release tumor antigens into circulation, potentially increasing the immunogenicity of the tumor and improving the effectiveness of immune checkpoint blockades [27].

Early clinical trials and retrospective studies suggest that patients who undergo surgical resection followed by immunotherapy may experience prolonged survival and better disease control [28]. Moreover, neoadjuvant immunotherapy is being investigated for its potential to downstage tumors, making them more resectable and reducing the risk of recurrence [29].

While the combination of surgical procedures and immunotherapy holds promise, challenges remain, including determining the optimal timing and sequencing of these treatments, managing potential adverse effects, and identifying biomarkers to predict response. 

## 6. Predictive Biomarkers

As previously mentioned, the benefit of immunotherapy in NENs remained limited, and not all patients with the same primary origin or extension experience the same response. This highlights the need to identify predictive biomarkers for patient subgroups that would benefit from this treatment strategy.

### 6.1. PD-L1

The programmed death receptor-1 (PD-1)/programmed death-ligand 1 (PD-L1) pathway has been recognized as crucial for tumors to evade the immune response. PD-L1 expression has been proposed as a predictive biomarker of response to immunotherapy [1]. Consequently, PD-L1 quantification and its implications have been investigated in NENs of different grades and origins.

PD-L1 expression has been associated with high-grade (G3) neoplasms [30]. In a cohort reported by Kim et al., including 15 G2 NENs and 17 G3 NENs, no patients in the first group and 41% of G3 NENs were considered PD-L1-positive (≥1% membranous expression). PD-L1 positivity was significantly associated with undifferentiated NENs [31]. In an independent series, PD-L1 expression was investigated in 68 NEN samples from different origins (37% GEP) with a high proliferation rate (Ki-67 above 20%). The authors found PD-L1 expression in 31.6% of G3 NENs. The proportion of PD-L1-positive tumors was similar across different primary sites but varied based on tumor differentiation (none of the three well-differentiated NENs showed positive expression) and disease extent (more common in patients with locally advanced disease at diagnosis) [32].

In a series of 70 small bowel (sb) NEN specimens (67.1% from primary tumors, 32.9% from metastases), the percentage of PD-L1 positivity (≥5% membranous expression) was found in 12.8% of tumor cells and 24.3% of tumor-infiltrating lymphocytes. Most of the NENs were grade 1 (67.1%) or grade 2 (32.86%), with a median Ki-67 of 2% [6]. Similarly, Da Silva et al. assessed PD-L1 and PD-L2 expressions in a series of well-differentiated GEP-NENs (64 sb-NENs and 31 PanNENs). 

PD-L1 positivity (≥5% membranous expression) was found in 12.8% of tumor cells and 24.3% of tumor-infiltrating lymphocytes. Most of the NENs were grade 1 (67.1%) or grade 2 (32.86%), with a median Ki-67 of 2% [6]. Similarly, Da Silva et al. assessed PD-L1 and PD-L2 expressions in a series of well-differentiated NENs.

PD-L1-positive expression (≥5% membranous expression) in tumor cells was low (0% of sb-NENs and 7.4% of PanNENs) [33]. Another series that quantified PD-L1 expression in surgical specimens from sb-NENs, which can provide a more accurate evaluation, found 39% positivity (≥1% membranous expression). Most of the samples were from ileal primary (87%) and grades 1–2 (92%) [34].

Although PD-L1 expression has been proposed as a plausible biomarker for prognosis and predicting improved survival in GEP-NENs [31,35,36], some limitations such as heterogeneous determination, the inclusion of primary and metastatic samples, and the unknown relationship between PD-L1 expression and other immune biomarkers can make data interpretation difficult.

### 6.2. Tumor Mutational Burden

As previously reported in various studies, the tumor mutational burden (TMB) tends to be low in well-differentiated neoplasms and higher in poorly differentiated carcinomas [37]. TMB has been proposed as a predictive marker for immunotherapy effectiveness [38]. Notably, the KEYNOTE-158 clinical trial (which included five patients with NENs) led to the approval of pembrolizumab for patients with a TMB of ≥10 mutations per megabase (mut/Mb), irrespective of the primary tumor site [39].

However, the universal TMB cut-off did not uniformly predict the same benefit across all tumor types. Possible explanations include the “quality” rather than the quantity of somatic mutations [40] and the dynamic biology with differences between primary tumors and metastatic sites [41].

For instance, a lower TMB of 1.09 mut/Mb in NENs compared to 5.45 mut/Mb in NECs has been reported in a cohort study of 69 NENs and 16 advanced NECs. Investigating the genomic alterations, the NEC cohort exhibited a greater frequency of InDels, structural variants, and polyploid genomes, suggesting a potentially more favorable landscape for immunotherapy when considering TMB as a single biomarker [42]. Similar findings have been replicated in independent cohorts, showing a higher TMB in G3 and NECs compared to well-differentiated NENs [43,44].

Like PD-L1 expression, TMB is not a perfect predictive biomarker of immunotherapy efficacy, but it is another component in the picture of the immune environment of NENs. In a multicenter phase Ib trial by Lu et al., patients with NENs (Ki-67 ≥ 10%) who had failed first line were treated with toripalimab. Among 40 patients, those with PD-L1 ≥ 10% or a high TMB (top 10%) showed a significantly higher ORR (50%), compared to those with PD-L1 expression < 10% (10.7%) and a low TMB (75% vs. 16.1%) [11].

Given the typically low TMB observed in NENs and the potential of the TMB as a biomarker for predicting immunotherapy responses, exploring sequential chemotherapy to induce an increase in the TMB before initiating immunotherapy warrants further investigation [45].

### 6.3. Immune Cell Infiltration in the Tumor

The presence of immune cells within the neuroendocrine neoplasm (NEN) tumor microenvironment (TME) is pivotal. The TME, which interacts dynamically with tumor cells, influences tumor regression, progression, and response to therapy [46]. The predominant immune cell subgroups extensively studied in the NEN TME include B and T lymphocytes, along with regulatory T cells (Tregs).

Overall, immune cell infiltration appears more pronounced in PanNENs compared to midgut carcinoids [33]. Da Silva et al. reported T cell infiltration in 68% of 87 PanNEN samples, which did not correlate with tumor grade. Takahashi et al. assessed immune cell infiltration across low-grade (*n* = 32, 61%), intermediate-grade (*n* = 15, 29%), and high-grade PanNENs (*n* = 3, 6%), as well as PanNECs (*n* = 2, 4%) [47]. Their findings indicated that PanNENs exhibited a ‘cold’ immune microenvironment characterized by limited tumor-infiltrating lymphocytes (TILs), whereas high-grade tumors showed abundant TILs [47]. In a cohort of 22 NENs from various primary sites with Ki-67 ≥ 20%, CD3+ T lymphocytes were present in 45.5% of samples, while CD8+ cytotoxic T cells were detected in 18.2% of samples [32].

In a study involving 87 primary tumors and 39 liver metastases from G1/G2 NENs originating from various sites, CD3+ lymphocytes were observed in 68% of primary lesions and 97% of liver metastases [48]. Potential reasons could include progressive immune system activation during tumor progression and accumulation of mutations. Similarly, in patients with grade 2 PanNENs, low infiltration of CD3+ T cells significantly predicted recurrence after tumor resection [48]. Cai et al. documented prolonged disease-free survival (DFS) associated with high CD8+ T cell infiltration in PanNENs, whereas elevated peritumoral CD4+ T cells were linked to worse DFS outcomes [49].

Strong infiltration of lymphocytes near or around tumor cells, coupled with a low density of regulatory T cells (Tregs), has been linked to an enhanced responsiveness to immunotherapy [37]. In a study involving 102 primary sb-NENs of grades 1 and 2, intratumoral immune infiltration was observed in 66% of samples. Approximately one-fifth of these tumors exhibited ectopic lymph nodes with activated germinal centers, which correlated with PD-L1 expression ≥ 50% in tumor cells [34]. In another study by Lamarca et al., including 62 sb-NEN, infiltration of CD8+ lymphocytes was focal in 93% of cases. However, this percentage decreased to 4.29% when considering moderate expression (infiltrates interspersed among tumor cells). Lymphoid aggregates were present in 27% of tumors [6]. The biological significance of these tertiary lymphoid structures in NENs remains ambiguous.

CD4+ FoxP3+ T regulatory (Treg) cells are thought to play a crucial role in immune evasion within patients with cancer. The presence of FoxP3+ cells has been noted to be more prevalent in high-grade compared to low-grade PanNENs [48]. Additionally, higher levels of FoxP3+ Tregs have been linked to poorer prognosis in patients with PanNEN, likely due to their immunosuppressive effects [50]. In another series of NENs, Tregs were identified in 55% of G2/G3 tumors, whereas only 16% of G1 metastatic NENs showed intratumoral Tregs [48]. Among a cohort of 39 metastatic midgut NENs, circulating Treg cells were detected in 6.5% of patients (compared to 3.7% in healthy donors), with levels reaching 9.0% in patients with a high tumor burden [51].

However, gaining a deeper understanding of the immune composition within these diverse tumors is essential to elucidate the prognostic implications of these NEN characteristics. Further insight into the genomic profiles and the NEN tumor microenvironment could aid in stratifying these patients for immunotherapy.

### 6.4. Notch Signaling Pathway 

Notch pathway deregulation has been implicated in tumorigenesis, disease progression, therapeutic resistance, and the suppression of neuroendocrine differentiation. Specifically, DLL3 acts as an inhibitory ligand of the Notch receptor and plays a role in the tumorigenesis of NENs and NECs [52]. In normal tissues, DLL3 is typically expressed at minimal levels, whereas it is overexpressed in approximately 85% of small cell lung cancer (SCLC) cases [52,53]. This observation has led to the proposition of DLL3 as a new biomarker and potential therapeutic target in SCLC.

DLL3 expression has been detected in GEP, bladder, and cervical NENs [54,55,56]. It is notably upregulated in high-grade NENs and NECs [57]. In a cohort of 47 patients with GEP NENs, DLL3 was present in 76.9% of NECs, while it was absent in 5 patients with G3 NENs [54]. DLL3 expression showed a statistically significant association with negative Ga-PET/CT scans and poorer prognosis. Among 155 surgically resected lung NENs, high DLL3 expression was observed in 12.2% of typical carcinoids and 24.4% of atypical carcinoids [57]. While high DLL3 expression is commonly linked to NECs and advanced disease, and is negatively correlated with survival in most studies, its role as a prognostic factor has not been consistently reported [54,58].

In Ali et al.’s series, which included patients with resected lung NENs, DLL3 overexpression was associated with a moderate-to-high inflammatory infiltrate (65.6% vs. 27.7%) [57]. In contrast, some studies in SCLC patients found that DLL3 levels were lowest in the SCLC-inflamed subtype, characterized by the expression of numerous immune checkpoints and human leukocyte antigens [59]. Overall, a deeper understanding of the correlation between DLL3 expression and the immune system, inflammatory biomarkers, and whether DLL3 expression predicts the ICI response is essential.

## 7. Discussion

This narrative review outlines the landscape of clinical trials exploring the role of immunotherapy in NENs and highlights the main potential biomarkers. 

While the incidence of GEP NENs remains relatively low, it is expected to rise in the coming years. At the time of initial diagnosis, many patients presenting with advanced disease require palliative local therapies. The majority of NENs demonstrate poor responsiveness to chemotherapy, making the identification of new therapeutic strategies, such as immunotherapy, crucial [36].

To date, clinical trials evaluating the efficacy of pembrolizumab, avelumab, spartalizumab, and toripalimab as monotherapies for NENs have been reported. While immune checkpoint inhibitors have shown a safety profile consistent with their use in other neoplasms, their efficacy as monotherapy in unselected NENs has been modest, with no complete responses achieved [7,8,10,11,21].

Given these underwhelming results, combination strategies aimed at overcoming the immunosuppressive tumor microenvironment and enhancing the immune response have been investigated. Although immunotherapy combinations show potential for greater efficacy than single-agent treatments, this was not consistently confirmed, even in high-grade NENs. This inconsistency underscores the need for a more precise definition of high-grade categories (differentiating between poorly differentiated and G3 NENs), proliferation index classification, histological subtypes (NENs versus NECs), and overall study design [24].

Despite the investigation of various classical biomarkers (such as PD-L1, TMB, and immune infiltration) and novel biomarkers (such as DLL3) in patients with NENs treated with immunotherapy, no clear correlations have been established. There are significant doubts regarding their predictive and prognostic implications, and they are not recommended for use in clinical practice. It is important to note that most studies evaluating immune biomarkers in NENs are small, retrospective, and not centrally reviewed, thus requiring a cautious interpretation of their results [60].

NENs are inherently challenging due to significant molecular differences among subtypes. While immunotherapy may benefit some patients, comprehensive tumor molecular profiling is essential for selecting potential candidates. Furthermore, specific NEN trials should consider the following factors: primary origin, histological subtype, proliferation index, and molecular background. An attractive strategy currently under evaluation involves combining immunotherapy with bispecific tumor-targeting antibodies, cell therapy, and biological agents like oncolytic viruses to achieve a more accurate immune response [61]. 

Ideally, clinical trials should incorporate tumor molecular profiling to identify predictive biomarkers that can enhance efficacy. Achieving this goal will require an international collaborative effort.

## 8. Future Directions

Apart from checkpoint inhibitors, there is increasing interest in bispecific antibodies targeting tumors, adoptive transfer of genetically modified autologous T cells, and oncolytic viruses as promising advancements in cancer immunotherapy. Table 2 summarizes some of the most notable ongoing clinical trials involving immunotherapy for NENs.

## 9. Conclusions

Immune checkpoint inhibitor monotherapy has shown modest activity for unselected NENs. Although combination immunotherapies are potentially more effective than single agents, this has not been homogeneously confirmed, even in G3-NENs. Other immunotherapeutic strategies in development include bispecific antibodies and oncolytic viruses. 

The predictive significance of some biomarkers (including PD-L1 expression, TMB, and DLL3) has been explored, with inconclusive results. The heterogeneity of NENs presents challenges for translational research, but a better understanding of the interplay between local/systemic immunity and the host is essential to identify patients with NENs who may benefit from immunotherapy.

## Figures and Tables

**Table 1:** Clinical trials of immunotherapy in patients with NENs.

Clinical Trial	Treatment	Therapy	Primary Site	N	Main Outcomes
KEYNOTE-028 [7]	Pembrolizumab	Monotherapy	Advanced PD-L1+ carcinoids or PanNENs	25 GI-NETs and 16 PanNENs	ORR: 12.0% (GI-NENs) and 6.3% (PanNENs)
KEYNOTE-158 [8]		Monotherapy	Advanced, well-differentiated NENs	40 PanNETs, 43 GI-NETs and 14 lung NETs	ORR: 3.7%
AVENEC [9]	Avelumab	Monotherapy	Advanced G3 NECs	29 NENs from different origins (72.4% GEP)	ORR: 6.9%
Yao et al. [10]	Spartalizumab	Monotherapy	Advanced thoracic/GEP-NENs and GEP-NECs	31 GI-NETs, 33 PanNETs, 30 thoracic NETs and 21 GEP-NECs	ORR: 7.4% (NENs) and 4.8% (NECs)
Lu et al. [11]	Toripalimab	Monotherapy	Advanced NENs (Ki-67 ≥ 10%)	8 WD-NENs and 32 NECs; 9 PanNENs and 31 ePanNENs	ORR: 25% (WD-NENs) and 18.7% (NECs)
DART SWOG 1609 [12]	Nivolumab and ipilimumab	Dual therapy (anti-PD1–anti-CTLA4)	Advanced, any grade NENs (excluding PanNENs)	14 WD-NENs and 18 NECs; 45% GI-NENs and 18% lung NENs	ORR: 0% (WD-NENs) and 44% (NECs)
CA209-538 [13]	Nivolumab and ipilimumab	Dual therapy (anti-PD1–anti-CTLA4)	Advanced, any grade NENs	29 NENs; 90% had G2 or G3; 39% lung NENs and 23% GEP-NENs	ORR: 24%; 43% in PanNENs, and 33% for atypical lung carcinoids
DUNE [14]	Durvalumab and tremelimumab	Dual therapy (anti-PDL1–anti-CTLA4)	Cohort 1: G1/2 lung NETs—Cohort 2: G1/G2 GI-NETs—Cohort 3: G1/2 PanNENs—Cohort 4: G3 GEP-NENs	27 lung carcinoids, 31 grade 1-2 GI-NETs, 32 grade 1-2 PanNETs and 33 grade 3 GEP-NENs (91% NECs)	ORR: 11.1% (cohort 1), 0% (cohort 2), 6.3% (cohort 3) and 9.1% (cohort 4)
NICE-NEC trial [15]	Nivolumab and platinum-based chemotherapy	Dual therapy (anti-PD1–chemotherapy)	GEP or unknown origin G3 NENs	38 NENs; 68.4% NECs	ORR: 54.1%
Owen et al. [16]	Nivolumab and temozolomide	Dual therapy (anti-PD1–chemotherapy)	Advanced, any grade NENs	28 NENs; 3 PanNENs, 11 lung NENs, 11 GI-NENs, 1 head and neck NENs and 2 unknown NENs	ORR: 32.1% (lung vs. others, *p* = 0.020)
Halperin D et al. [17]	Atezolizumab and bevacizumab	Dual therapy (anti-PDL1–antiangiogenic)	Advanced, G1/2 NENs	40 NENs; 20 PanNENs and 20 ePanNENs	ORR: 20% PanNENs and 15% ePanNENs
CABATEN/GETNE-T1914 [18]	Atezolizumab and cabozantinib	Dual therapy (anti-PDL1–kinase inhibitor)	Cohort 1: WD lung NENs—Cohort 2: ATC—Cohort 3: ACC—Cohort 4: PPGL—Cohort 5: WD GEP NENs—Cohort 6: G3 extrapulmonary NENs	9 WD lung NENs, 14 ATC, 24 ACC, 13 PPGL, 24 WD GEP NENs and 9 G3 extrapulmonary NENs	ORR: 0% (cohort 1), 21.4% (cohort 2), 8.3% (cohort 3), 7.7% (cohort 4), 16.7% (cohort 5) and 0% (cohort 6)

ACC: adrenocortical carcinoma. ATC: anaplastic thyroid cancer. ePanNENs: extra-pancreatic neuroendocrine neoplasms. G: grade. GEP: gastro-entero-pancreatic. GI: gastrointestinal. NEC: neuroendocrine carcinomas. NENs: neuroendocrine neoplasms. ORR: overall response rate. PanNENs: pancreatic neuroendocrine neoplasms. PPGL: pheochromocytoma/ paraganglioma. WD: well differentiated.

**Table 2:** Ongoing clinical trials involving immunotherapy combination in NENs.

Clinical Trials.gov Identifier	Study Title	Phase	NEN Population	Treatment Arms	Primary Endpoint
03457948	Pembrolizumab With Liver-Directed or Peptide Receptor Radionuclide Therapy for Neuroendocrine Tumors and Liver Metastases	II	Advanced grade 1–2 NENs	- Arm 1: pembrolizumab + 177Lu-DOTATATE (4 sessions). - Arm 2: pembrolizumab + arterial embolization, 3–7 days following the first dose of pembrolizumab. - Arm 3: pembrolizumab + yttrium-90 microsphere radio embolization, 3–15 days following the first dose of pembrolizumab.	ORR
04525638	A Clinical Study to Assess the Combination of Two Drugs (177Lu-DOTATATE and Nivolumab) in Neuroendocrine Tumors	II	Advanced, well-differentiated grade 3 NENs or NECs of the pancreas, GI, lung, and unknown primary	All subjects will be treated with nivolumab + 177-Lu-DOTATATE (maximum 4 cycles).	ORR
05058651	Evaluating the Addition of the Immunotherapy Drug Atezolizumab to Standard Chemotherapy Treatment for Advanced or Metastatic Neuroendocrine Carcinomas That Originate Outside the Lung	II/III	Advanced extrapulmonary NECs	- Arm 1: atezolizumab + cisplatin/carboplatin + etoposide (4 cycles). Maintenance: atezolizumab. - Arm 2: atezolizumab + cisplatin/carboplatin + etoposide (4 cycles). Observation for 1 year. - Arm 3: cisplatin/carboplatin + etoposide (4 cycles). Observation for 1 year.	OS
04969887	Combination Immunotherapy in Rare Cancers Under InvesTigation (MOST-CIRCUIT)	II	Atypical bronchial carcinoid, NECs, and grade 3 NENs independent of primary site (SCLC excluded)	All subjects will be treated with nivolumab + ipilimumab at concurrently (4 cycles). Nivolumab until progression (up to 2 years).	6 months PFS and ORR
01174121	Immunotherapy Using Tumor Infiltrating Lymphocytes for Patients with Metastatic Cancer	II	Metastatic NENs	- Arm 1: CD8+-enriched TIL—cyclophosphamide and fludarabine (C+F) + young CD8+-enriched TIL + high-dose aldesleukin. - Arm 2: unselected TIL—C+F + young, unselected TIL + high-dose aldesleukin. - Arm 3: unselected TIL—C+F + young, unselected TIL + high-dose aldesleukin + pembrolizumab prior to cell administration and 3 additional doses following cell infusion. - Arm 4: unselected TIL—C+F + young, unselected TIL + high-dose aldesleukin + pembrolizumab within 4 weeks of progressive disease (for up to 8 doses).	ORR
03412877	Administration of Autologous T-Cells Genetically Engineered to Express T-Cell Receptors Reactive Against Neoantigens in People with Metastatic Cancer	II	NENs refractory to second-vb line therapy	- Arm 1: cyclophosphamide and fludarabine + individual patient TCR-transduced PBL + high- or low-dose aldesleukin. - Arm 2: cyclophosphamide and fludarabine + individual patient TCR-transduced PBL + high- or low-dose aldesleukin + pembrolizumab prior to cell administration and 3 additional doses following cell infusion.	ORR
05882058	A Study to Test Whether Different Doses of BI 764532 Help People with Small Cell Lung Cancer or Other Neuroendocrine Cancers	II	SCLC, epNECs, LCNEC	Drug: BI 764532 (DLL3/CD3 T cell engaging bispecific antibody—two different doses).	OR
04702737	A Study of AMG 757 in Participants with Neuroendocrine Prostate Cancer	I	Neuroendocrine prostate cancer	Drug: tarlatamab (bispecific T-cell engager molecule, binds both DLL3 and CD3—dose exploration/expansion).	Security
5652686	A Phase 1 Study of PT217 in Patients with Advanced Refrac-tory Cancers Expressing DLL3	I	LCNEC, neuroendocrine prostate cancer and GEP-NEC	Drug: PT217 (bispecific antibody (bsAb) against human DLL3 (huDLL3) and human CD47 (huCD47))	Security
5879978	A Study to Test How Well Different Doses of BI 764532 in Combination with Ezabenlimab Are Tolerated by People with SCLC and Other Neuroendocrine Tumos That Are Positive for DLL3	I	LCNEC and NEC of any origin	Drug: BI 764532 (a DLL3)/CD3 IgG-like T-cell engager) Drug: ezabenlimab (anti-PD-1 mAb)	MTD
4471727	Study in Patients with Advanced Cancers Associated with Expression of DLL3	I/II	Neuroendocrine prostate cancer, high grade neuroendocrine tumor types other than SCLC	Drug: HPN328 (tri-specific DLL3-targeting T-cell engager—dose exploration/expansion) Drug: atezolizumab (combination)	Security
4429087	A Study to Test Different Doses of BI 764532 in Patients with SCLC and Other Neuroendocrine Tumours That Are Positive for DLL3	I	LCNEC and NEC	Drug: BI 764532 (a DLL3)/CD3 IgG-like T-cell engager)	MTD

epNECs: extra-pulmonary neuroendocrine carcinoma. GEP-NEC: gastroenteropancreatic neuroendocrine carcinoma. LCNEC: large cell neuroendocrine carcinoma. MTD: maximum tolerated dose. NECs: neuroendocrine carcinomas. NENs: neuroendocrine neoplasms. OR: objective response. ORR: overall response rate. OS: overall survival. PFS: progression-free survival. SCLC: small cell lung cancer.

## Data Availability

No new data were created or analyzed in this study. Data sharing is not applicable to this article.
